# Combining Different Tools for EEG Analysis to Study the Distributed Character of Language Processing

**DOI:** 10.1155/2015/865974

**Published:** 2015-12-02

**Authors:** Armando Freitas da Rocha, Flávia Benevides Foz, Alfredo Pereira

**Affiliations:** ^1^Research on Artificial and Natural Intelligence (RANI), Rua Tenente Ary Aps 172, 13207-110 Jundiaí, Brazil; ^2^CEFAC-Saúde e Educação, Rua Anchieta 670, Sala 22, 13201-804 Jundiaí, SP, Brazil; ^3^Department of Education, Institute of Biosciences, University of São Paulo, Campus Rubião Jr., 18618-970 Botucatu, SP, Brazil

## Abstract

Recent studies on language processing indicate that language cognition is better understood if assumed to be supported by a distributed intelligent processing system enrolling neurons located all over the cortex, in contrast to reductionism that proposes to localize cognitive functions to specific cortical structures. Here, brain activity was recorded using electroencephalogram while volunteers were listening or reading small texts and had to select pictures that translate meaning of these texts. Several techniques for EEG analysis were used to show this distributed character of neuronal enrollment associated with the comprehension of oral and written descriptive texts. Low Resolution Tomography identified the many different sets (*s*_*i*_) of neurons activated in several distinct cortical areas by text understanding. Linear correlation was used to calculate the information *H*(*e*_*i*_) provided by each electrode of the 10/20 system about the identified *s*_*i*_. *H*(*e*_*i*_) Principal Component Analysis (PCA) was used to study the temporal and spatial activation of these sources *s*_*i*_. This analysis evidenced 4 different patterns of *H*(*e*_*i*_) covariation that are generated by neurons located at different cortical locations. These results clearly show that the distributed character of language processing is clearly evidenced by combining available EEG technologies.

## 1. Introduction

Recent studies using functional magnetic resonance imaging (fMRI), magnetoencephalography (MEG), and electroencephalography (EEG) have expanded our knowledge about the neural circuits enrolled in language comprehension and production by demonstrating that these cognitive activities involve a large number of areas, in addition to Broca's and Wernicke's areas (e.g., [1–21]). Neurons at distinct cortical areas seem to play specific roles in speech processing but either verbal understanding or production may be explained as resulting from activity of just one specific area, for example, Wernicke or Broca. The large number of different types of neurons involved in language understanding and production, as well as the complex dynamics of their relations, points to the distributed character of language processing. The theory of distributed intelligent processing systems (DIPS) introduced by Artificial Intelligence researchers as a formal theory of intelligence has been applied to model brain activity of cognitive functions (e.g., [22, 23]).

To register and to study DIPS's activity, a careful choice of how to collect data and statistical tools is required, due to the large number of distinct agents that enroll to intelligently solve a cognitive task and due to the complex interactions established by them to handle the distinct subtasks of a complex cognitive function. In case of brain, it is necessary to select tools having high temporal discrimination to capture details of the complex neuronal interplay as well as having suitable spatial discrimination to identify the main actors of such interplay. Electroencephalography (EEG) allows registering brain activity at a range of milliseconds that is specified by sampling frequency. In addition, recent development highly improved its spatial discrimination, despite restricting analysis to cortical neurons. Because of this, Rocha et al. [24] proposed EEG as the tool of choice for investigating human cognition if all recent developed statistical tools for EEG analysis are applied to analyze recorded activity associated with cognition. This is the approach used in this paper to investigate understanding of both oral and written texts.

*Distributed Intelligent Systems.* The theory of Distributed Intelligent Processing System (DIPS) was first developed in the field of Artificial Intelligence to formalize those systems comprised of multiple agents that individually have some sort of expertise in solving defined problems, while if they work together they may solve tasks of higher complexity. DIPS intelligence is a function of the types of tools used by its agents, as well as of how and for what purpose these tools are used [25–31]. The large number of different types of neurons enrolled in language understanding and production revealed by the neuroscience literature cited above speaks in favor of a distributed character for language processing, a fact that was recently acknowledged in the literature (e.g., [17, 32]).

Central to the concept of a DIPS is the proposal that reasoning is supported by the cooperative activity of a collection of agents, each having specific knowledge or tool useful in handling a complex task that is of interest to the whole system [23, 31]. Agents enroll or are recruited to support reasoning if their knowledge or ability may contribute to the handling of the task in question. The same agents may contribute to the solution of different tasks but different types of reasoning have also to recruit distinct types of agents. For example, listening and reading are supposed to enroll common sets of agents involved in syntactic and semantic analysis, but they also have to include different sets of agents involved in acoustic and visual analysis of sensory input.

In case of DIPS, no component plays the role of the knowledge or data storage center. Part of DIPS knowledge relies on agent specialization and part of them is encoded by relations shared by their agents. Relationship among agents has to be easily modified whenever necessary to support learning. Language cognition is part determined by agent specialization in handling sensory and motor systems involved in phoneme and grapheme analysis and production, but syntax and semantics are dependent on established relations among agents, most of them specialized in other aspects of cognition.

Any DIPS control is logically and geographically distributed. Control is not a property of specific agents, but it is embedded in the rule for message passing among agents. Messages are exchanged directly because agents are directly connected (mailing address systems) or by means of blackboard agents (working memory systems). Agents may enroll in reasoning attending messages posted on blackboards or are recruited by agents that know about their abilities. Message exchange results in oscillatory activity between sets of agents.

Sets of different agents may propose different task solutions depending on their knowledge and ability and because of this conflict is a common occurrence in DIPS reasoning. Sets of neurons specialize in checking coherence of different hypotheses while others enroll in conflict solving. Solution of complex tasks requires recurrent cycles of processing, when those agents less likely to contribution to task solution are disconnect and results obtained so far are consolidated.

*Experimental Lines of Evidence of the Distributed Character of Language Processing.* Many studies in the literature address some specific topics of the DIPS character of language processing as pointed out by Price [17] who, after reviewing language fMRI literature for the last 20 years, concluded that “the different language functions are not localized in specific brain regions, but they were distributed across networks of regions with each area making a specific contribution to performance of the task which depends on its connections to other areas in a parallel distributed hierarchy.”

For example, Brennan and Pylkkänen [2] studied the time course and spatial distribution of brain activity associated with sentence processing and found an increased anterior temporal activity for sentences compared to word lists, which started approximately 250 ms after word onset. They also observed increased activation in a network of other brain areas, extending across the posterior temporal, inferior frontal, and ventral medial areas.

In addition, Laaksonen et al. [11] identified the spatiotemporal patterns of task effects in three MEG data sets, all variants of a picture naming task. They concluded that evoked responses and rhythmic modulation yielded largely separate networks, with spatial overlap mainly in the sensorimotor and primary visual areas. Moreover, in the cortical regions that were identified with both measures, the experimental effects they conveyed differed in terms of timing and function. Their results suggest that the two phenomena are largely detached and that both measures are necessary for an accurate portrayal of brain activity to identify all different electrical sources *s*_*i*_ associated with language processing.

In this line of approach, Obleser and Kotz [16] reported that listening to speech under adverse conditions triggers different evoked responses: (a) N100 component to a degraded sentence's onset that correlates with participants' comprehension scores, but usually more vigorous for more degraded sentences, and (b) pronounced N400 in response to low-close sentence-final words that increases linearly with improving speech intelligibility, reflecting the integration* effort* of words into context. In addition, they observed transient enhancement in *γ*-band power (*γ*, ~40–70 Hz) during high-close sentence-final words (~600 ms) that reflect top-down-*facilitated* integration and a negative correlation of N100 amplitude at sentence onset; the later *γ*-band response is found in moderately degraded speech. This *γ*-band effect also varies parametrically with signal quality.

This kind of observations supports the proposal that neuronal oscillations define short temporal windows for flexible communication between widely distributed neuronal ensembles [17, 33]. Although broad synchronization in distributed processing systems is dependent on the action of specific circuits, as in thalamus-cortical synchronization [34], short term communication is dependent on interconnections between the sets of neurons located in different brain areas supporting transient functional couplings [17].

Although controversies exist, semantic/conceptual processing during language comprehension has traditionally been associated with N400, whereas syntactic processing is generally thought to correlate with a parietal positive Event Related Potential (ERP) effect, the so-called P600 (e.g., [13, 35–38]). Such classic language ERP components may be considered as linked to the cyclic character of language processing, signaling the dynamic of each processing cycle involved in solving specific reasoning subtasks. According to Bastiaansen and Hagoort [1], neuronal synchrony is a mechanism by which the brain integrates the different types (phonological, orthographic, syntactic, and semantic) of information about language. In addition, Giraud and Poeppel [6] proposed that neuronal oscillations are ubiquitous in the brain and may contribute to cognition in several ways, for example, by segregating information and organizing spike timing. In the case of speech and language processing, they proposed that neuronal oscillations organize incoming information into units of the appropriate temporal granularity.

Hasson et al. [39] remarked that, during speech communication, two or more brains are coupled through an oscillatory signal, and the speech signal across all languages and contexts has its own amplitude modulation at rhythms ranging between 3 and 8 Hz. This rhythm corresponds roughly to the timescale production of 3 to 8 syllables per second. Because recent theories of speech perception note that the amplitude modulations in speech closely match the structure of the 3–8 Hz theta oscillation, they suggested that the speech signal could be coupled and/or resonate (amplify) with ongoing oscillations in the auditory regions of a listener's brain.

*Identifying Language Cortical Agents.* Low Resolution Tomography (sLORETA) has been used to study many distinct characteristics of neural language processing and has contributed to the understanding of the DIPS character of language processing by helping to identify all different electrical sources *s*_*i*_ associated with language processing.

Adorni and Proverbio [40] studied the timing and topographical distribution of ERP components associated with word/nonword discrimination using LORETA for ERP source location. They demonstrated that words were discriminated from pseudowords because larger N2 responses to words than to pseudowords were observed over the left occipitotemporal areas at 300 ms after stimulus. Concrete words and abstract words were discriminated as early as 350 ms after stimulus, with larger responses to concrete than to abstract words over the mesial occipital regions. Concreteness-related ERP differences were also observed in the amplitudes of the anterior later positive component (LP), between 370 and 570 ms, with larger responses to abstract words than to concrete words. These authors concluded that words (both abstract and concrete) were associated with stronger activation of the left fusiform gyrus and the left temporal cortex compared to pseudowords. Concrete word processing was associated with stronger activation of the left extrastriate visual areas (namely, BA 18 and BA 19) compared to abstract word processing.

Lavric et al. [41] studied neural activity associated with the generation of regular and irregular past tense and used LORETA for ERP source location. A data-driven algorithm temporally segmented the ERPs into 16 distinct epochs of stable field configuration (microstates). A space-oriented brain electric field analysis determined that one epoch, 288–321 ms after the verb presentation, demonstrated significant differences between the regular and irregular verb conditions. In addition, they found that this microstate was more active for regular conditions in the right prefrontal and right temporal areas and for irregular conditions in the left temporal areas and the anterior cingulate cortex.

Yang et al. [42] studied comprehension of different types of Chinese (Mandarin) relative clauses (object versus subject-extracted) to test the universality and language specificity of sentence comprehension processes using ERPs and LORETA. LORETA source localization showed activation of posterior dominance (e.g., BA 22/39/19/41/42), which supports the integration of structure mapping (P600) and meaning derivation (N400) in a developing sentential representation. More left-lateralized anterior regions of a frontal-temporal network (e.g., BA 47/38) became active later in the sentence when the thematic-role specification for multiple referents may have required additional cognitive and memory resources.

Ishiwatari et al. [43] studied EEG activity associated with silent reading of words in different scripts: kanji (Japanese logograph), hiragana (a Japanese syllabogram), and English. ERP waveforms and 2D LORETA topographic maps revealed that, independently of the scripts, the silent word reading process was comprised of three distinct phases reflected in N150, P200, and the late positive component (LP), respectively. These results suggested that reading words processed in a language with a writing system that differs from the native language is quite different in processing time course.

*Quantifying Information Provided by EEG Recording.* The signal (*v*(*e*_*i*_, *t*)) that is recorded by the electrode *e*_*i*_ is a weighted *w*(*e*_*i*_, *s*_*l*_) sum of the dendritic activities at innumerous cortical locations (*s*_*l*_) resulting from synchronous excitatory and inhibitory inputs (e.g., [34]). Because of this, the correlation coefficient *r*_*i*,*j*_ calculated between the recorded electrical activities *v*(*e*_*i*_, *t*) and *v*(*e*_*j*_, *t*) recorded by *e*_*i*_, *e*_*j*_ is expected to be highly dependent on the weights (*w*(*e*_*i*_, *s*_*l*_)) determining the contribution of each *s*_*l*_ to these recorded activities. If *w*_*i*_^*l*^, *w*_*j*_^*l*^ are high, then source *s*_*l*_ is an important determinant of both *v*(*e*_*i*_, *t*) and *v*(*e*_*j*_, *t*) increasing the determination coefficient *r*_*i*,*j*_ whenever it is active. If two different sources *s*_*l*_, *s*_*m*_ are influential upon *e*_*i*_, *e*_*j*_, respectively, then *r*_*i*,*j*_ approaches 1 or −1 if they are positively or inversely correlated. In this context, the determination coefficient |*r*_*i*,*j*_| increases if *s*_*l*_, *s*_*m*_ are either near to both *e*_*i*_, *e*_*j*_ or are synchronized. In contrast, if all sources that are influential upon *v*(*e*_*i*_, *t*), *v*(*e*_*j*_, *t*) are silent, then |*r*_*i*,*j*_| approaches 0.5. In this theoretical context, the highest uncertainty about the information provided by *e*_*i*_, *e*_*j*_ about *s*_*l*_ and *s*_*m*_ occurs when |*r*_*i*,*j*_| approaches 0.5, and it is minimum when |*r*_*i*,*j*_| approaches 1 or 0.

Taking these considerations into account, Rocha et al. [22–24, 44] proposed that the amount of information *H*(*e*_*i*_) provided by *e*_*i*_ about the sources *s*_*l*_ is a function of |*r*_*i*,*j*_|. In this line of reasoning, if the sources *s*_*l*_ contributing to *v*(*e*_*i*_, *t*) are strongly activated and/or synchronized, then *H*(*e*_*i*_) increases proportionally; otherwise, it approaches 0.

While Event Related Activity (ERA) and Spectral Band Analysis (SBA) may provide information about specific and localized sources *s*_*l*_ involved in task solving, *H*(*e*_*i*_) provides information about the spatial and temporal distribution of these sources and, therefore, provides information about how different sets of neurons enroll themselves in a widely distributed network to solve a task [23]. Another interesting *H*(*e*_*i*_) property is that it summarizes information about all sources *s*_*l*_ into a single variable, simplifying many analyses (e.g., regression analysis, Principal Component Analysis) involving behavioral and neural variables [23, 44].

*Multivariate Analysis.* Principal Component Analysis (PCA) is a statistical tool to investigate patterns of covariation in a large number of variables and to determine whether information may be condensed into small sets of these variables called principal components [45]. This transformation is defined in such a way that the first principal component is the one that accounts for as much of the variability in the data as possible, and each succeeding component in turn explains the subsequent amount of variance possible under the constraint that it be orthogonal to (i.e., uncorrelated with) the preceding components. *H*(*e*_*i*_) PCA condenses information provided by all recording electrodes *e*_*i*_ about the sources *s*_*l*_ involved in a cognitive task into a set of components *P*_*j*_ according to *H*(*e*_*i*_) covariation. In this context, each *P*_*j*_ provides the sets *s*_*l*_ of neurons that enroll together in cognitive task variables [23, 24, 44]. For such a purpose, PCA mappings are constructed taking into account the loading values *f*_*j*_(*e*_*i*_) of *H*(*e*_*i*_) on each of the components *F*_*j*_, in order to represent the activity of the neural circuits enrolled in a cognitive task.

*Combining Technologies to Study Listening and Reading.* The purpose of the present study was to use the available techniques for EEG analysis to study brain activity associated with text listening and reading assuming that language cognition is supported by distributed intelligent reasoning as discussed above.

**Figure 1:**
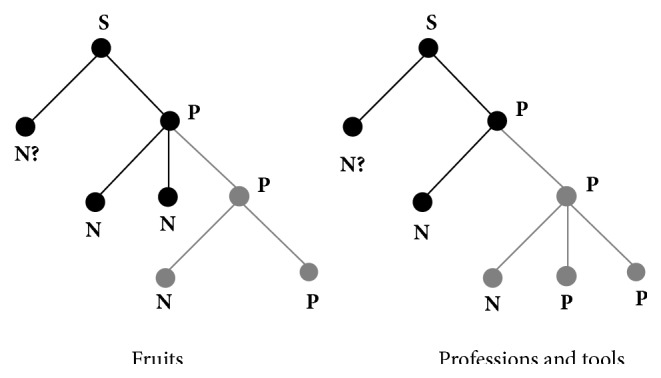
The two syntactic structures of the used texts.** S**: sentence;** N**: nominal component;** P**: predicate component.

**Figure 2:**
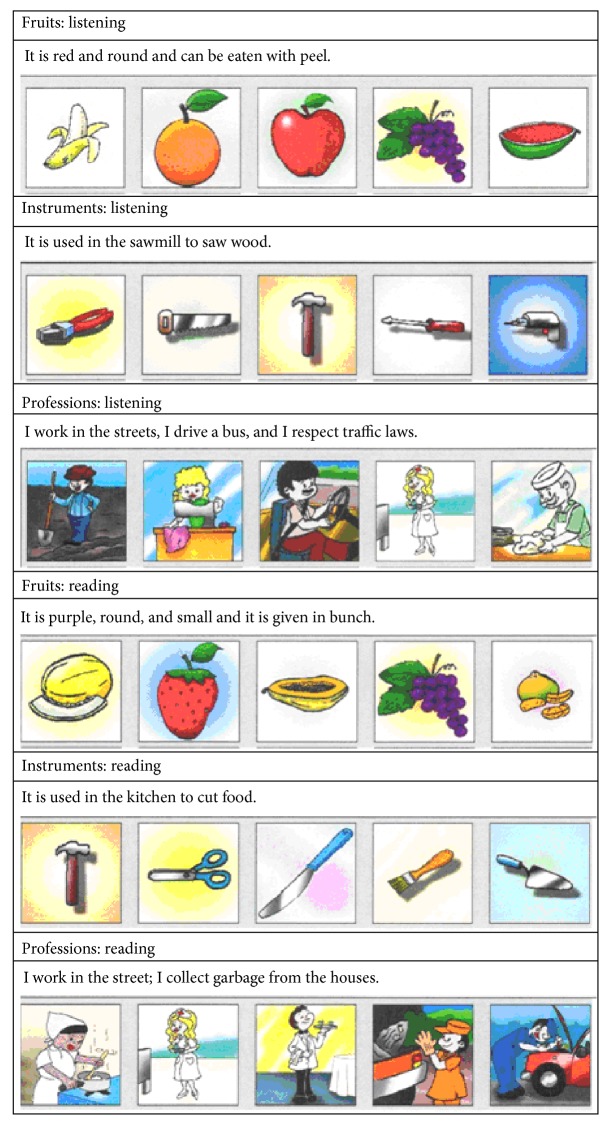
Examples of listening and reading tasks about fruits, tools, and professions.

**Figure 3:**
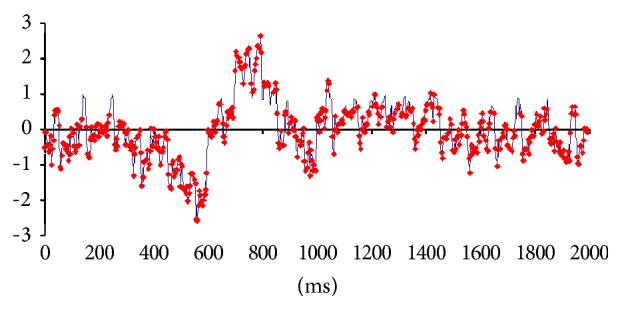
Grand average calculated for visual epoch** V**_**R**_ of reading task. Red dots mark the EEG moments that were used for LORETA calculations because their *Z* scores were above 1.961.

Texts used in this investigation described characteristics of fruits, the function of instruments, and the place and function associated with professions (Figures 1, 2, and 3). After the text was played in listening tests or displayed in reading tests, a set of figures was provided for the volunteer to select the adequate meaning of the text. It is supposed here that the volunteer kept verbal information acquired during the listening or reading epoch in memory, to select the figure that best corresponds to verbal decoding. In this way, each text processing involves a verbal phase associated with listening (**L**) or reading (**R**) activities and a visual phase analysis (**V**_**L**_ or** V**_**R**_) of the figures.

It is supposed, here, that a large number of cortical areas (*s*_*l*_) will be identified by sLORETA as sources for the recorded event related activity (ERA) and cortical oscillations (Band Frequency Analysis, BFA) during both the verbal (**L **and** R**) and visual (**V**_**L**_ and** V**_**R**_) phases. Results are expected to reveal a very complex temporal and spatial distribution of these sources *s*_*l*_, most of them being located at similar cortical areas for both listening and reading processing but some of them being specifically associated with one of these activities.

*H*(*e*_*i*_) PCA is expected to disclose different components *P*_*j*_ explaining the complexity of *s*_*l*_ temporal and spatial distribution by showing how neurons widely spread over the cortex enroll themselves to support neural activity associated with** L**,** R**,** V**_**L**_, and** V**_**R**_.

If such goals are attained, then a strong case is made for considering language processing as a cognitive function supported by a distributed intelligent processing system instead of by specific neurons located in a small number of areas.

## 2. Methods

### 2.1. Population

Volunteers were students of the university that spontaneously attended an advertisement about the research posted on campus. No money or course credit incentives were promised or provided. The purpose of the research was explained, and 20 males (age: 20 ± 0.7) and 21 females (age: 20 ± 0.3) agreed to participate in the EEG recording session. All participants were right-handed, monolingual, and native Portuguese speakers.

This study was approved by the Ethics Committee for Research Projects Analysis (CAPPesq) and the Hospital das Clinicas, School of Medicine, University of São Paulo (HCFMUSP), under protocol number 117/00.

### 2.2. Procedure

A fluent Portuguese native speaker was tape-recorded while reading aloud the written form of the 30 texts composed of short sentences (**S**) about unnamed (**N?**) fruits, tools, or professions having the syntactic structure that may be formalized by means of theory of Formal Grammars [29, 30, 46] as illustrated in Figure 1. According to this theory, any sentence** S** of a given language is understood as a set of relations among classes of symbols, for example, Noun (**N**) and Predicates (**P**) with** P** being recursively decomposed into other** N**'s and** P**'s. Texts used in the present experiment have one of the two structures displayed in Figure 1.

Texts provide information about all** N**'s and** P**'s except** N?**. Using these pieces of information, the volunteer was asked to provide** N?** by selecting one of 5 figures. All texts provided information (black in Figure 1) to trigger thinking about possible** N?**'s and additional information (grey in Figure 1) to increase the probability of one of these alternatives as task solution. Texts have, therefore, two syntactical components: triggering and solving components.

The texts were randomly separated into two different sets of 15 tests to be used in the listening or reading tasks. After listening or reading the text, volunteers had to select one among 5 pictures related to text subject (see Figure 2) as the best semantic matching to** N?**. Volunteers were given as much time as they needed to solve the task.

### 2.3. EEG Recording

EEG was recorded (20 electrodes placed according to the 10/20 system; impedance smaller than 10 Kohm; notch filter 50 Hz; sampling rate of 256 Hz and 10-bit resolution, ear lobe reference) while the volunteers were solving the text.

The exact time when the text (*t*_*t*_) and figures (*t*_*f*_) became available and the time (*t*_*d*_) when the volunteers chose an answer, that is, made a decision, were registered in the experimental data base together with information about the type of answer (right or wrong). Response time was calculated as *t*_*d*_ − *t*_*f*_ for all activities and volunteers.

The following EEG epochs were selected for analysis:

(1) Two seconds of duration following each test onset *t*_*t*_ were selected for analysis and denoted here as epoch** L** in the case of the listening task and as epoch** R** in the case of the reading task.
(2) Two seconds of duration following *t*_*f*_ were selected for analysis and denoted here as epoch** V**_**L**_ in the case of the listening task and as epoch** V**_**R**_ in the case of the reading task.

Each volunteer made 15 decisions about the meaning of the texts they listened to and made 15 decisions about the texts they read. Therefore, a total of 15 EEG epochs associated with listening decoding and 15 EEG epochs associated with** L**,** R**,** V**_**L**_, and** V**_**R**_ were analyzed for each volunteer. Therefore, the total number of selected epochs was 1320 (number of volunteers *∗* number of epochs *∗* number of tests). Bad EEG records were discarded. A total of 1155 EEG epochs were used for analysis with a rejection rate of 12.5%.

### 2.4. sLORETA

sLORETA uses measurements of scalp electric potential differences (EEG) or extracranial magnetic fields (MEG) to find the 3D distribution of generating electric neuronal activity with exact zero error localization to point-test sources [47]. Here, sLORETA was used for localizing the possible EEG source generators different sets (*s*_*l*_) associated with the EEG epochs** L**,** R**,** V**_**L**_, and** V**_**R**_.

The corresponding** L**,** R**,** V**_**L**_, and** V**_**R**_ epochs were averaged for each electrode and for all volunteers into different files, generating the corresponding EEG averaged files of each experimental epoch. Therefore, each of these files was composed of the corresponding EEG averages calculated for each of the 20 electrodes used to record the electrical activity associated with 1155 experimental epochs. A grand average was calculated for each of the above files, and the corresponding *Z* score was calculated for each of the 512 moments; the EEG was sampled at the rate of 256 Hz during the 2 seconds of duration of each epoch. Only those EEG moments with a *Z* score greater than 1.961 (5% of significance level) were selected for LORETA EEG source identification (e.g., Figure 3). LORETA software may provide more than one solution for each of these moments, but it orders these solutions according to its statistical methods. Here, only those areas provided by the first LORETA solution were assumed as possible source generators *s*_*l*_ for the studied EEG epochs.

LORETA software was also used to calculate the cross-spectra (CS) and the time varying cross-spectra (TVCS) for the averaged sets** L**,** R**,** V**_**L**_, and** V**_**R**_ epochs. We used tools from the LORETA software package to calculate TVCS for the entire (2 seconds) duration of experimental epochs. There is no specific rule to determine the size of this window; here, its duration was set ad hoc to 100 ms. Both CS and TVCS were calculated for frequencies from 0.5 to 100 Hz.

### 2.5. Measuring the Amount of Information *H*(*e*_*i*_) Provided by Each *e*_*i*_ about the EEG Sources *s*_*l*_

Correlation analysis of EEG activity *v*_*i*_(*t*) recorded by the different electrodes *e*_*i*_ may be used to summarize information provided by each electrode *e*_*i*_ about all involved sources *s*_*l*_ into a single variable *H*(*e*_*i*_) as proposed by Rocha et al. [22, 23, 44]. The rationality is the following.

Pearson's correlation *R* is +1 in the case of a perfect positive linear relationship (correlation), −1 in the case of a perfect negative linear relationship (anticorrelation), and some value between −1 and +1 in all other cases, indicating the degree of linear dependence between the variables. As it approaches zero, there is less relationship (closer to uncorrelated). The closer the coefficient is to either −1 or 1, the stronger the correlation is between the variables. The correlation strength *r* is defined as |*R*|.

Because *v*_*i*_(*t*) is a weighted summation of the electrical currents generated by each *s*_*l*_, *r*_*i*,*j*_ calculated between the activities *v*_*i*_(*t*) and *v*_*j*_(*t*) recorded by *e*_*i*_, *e*_*j*_ is expected to be highly dependent on *w*_*i*_^*l*^, *w*_*j*_^*l*^ weights determining the contribution of *s*_*l*_ to these recorded activities. If *w*_*i*_^*l*^, *w*_*j*_^*l*^ are high, then source *s*_*l*_ is an important determinant of both *v*_*i*_(*t*) and *v*_*j*_(*t*) increasing the determination coefficient *r*_*i*,*j*_ whenever it is active. If two different sources *s*_*l*_, *s*_*m*_ are influential upon *e*_*i*_, *e*_*j*_, respectively, then *r*_*i*,*j*_ approaches 1 or −1 if they are positively or inversely correlated. In this context, the determination coefficient |*r*_*i*,*j*_| increases if *s*_*l*_ is active and/or *s*_*l*_, *s*_*m*_ are synchronized and active. In contrast, if all sources that are influential upon *v*_*i*_(*t*), *v*_*j*_(*t*) are silent, then |*r*_*i*,*j*_| approaches 0.5.

In this context, the highest uncertainty about the information provided by *e*_*i*_, *e*_*j*_ about *s*_*l*_ and/or *s*_*m*_ occurs when |*r*_*i*,*j*_| approaches 0.5, and it is minimum when |*r*_*i*,*j*_| approaches 1. Therefore, in the same line of reasoning used by Shannon [48] to define the amount of information provided by a random variable, it was proposed [22, 23, 44, 49] that the* informational equivalence H*(*r*_*i*_, *r*_*j*_) of *v*_*i*_(*t*), *v*_*j*_(*t*) recorded by *e*_*i*_, *e*_*j*_ is the expected value *E*(*I*(*r*_*i*,*j*_)) of the information *I*(*r*_*i*,*j*_) provided by |*r*_*i*,*j*_|.

In this line of reasoning, the information *H*(*e*_*i*_) provided by electrode *e*_*i*_ about the sources *s*_*l*_ activated for task solution is calculated from all *H*(*r*_*i*_, *r*_*j*_) in comparison to $H(\bar{r_{i}})$. If *H*(*r*_*i*_, *r*_*j*_) is equal to the mean information $H(\bar{r_{i}})$ provided by all other (19) electrodes *e*_*j*_, then *v*_*i*_(*t*) compared to *v*_*j*_(*t*) did not reduce the uncertainty about *s*_*l*_ being involved in the task solution. In contrast, if *H*(*r*_*i*_, *r*_*j*_) approaches zero, then all fundamental sources *s*_*l*_ for the activity recorded *e*_*i*_ are most likely involved in the task solution. In this line of reasoning, *H*(*e*_*i*_) measures the information provided by the electrode *e*_*i*_ about all sources *s*_*l*_ activated by a given cognitive activity, and it is calculated as previously reported [23, 44].

### 2.6. Principal Component Analysis and *H*(*e*_*i*_) Covariation

Principal Component Analysis (PCA) is a statistical tool to investigate patterns of covariation in a large number of variables and to determine whether information may be condensed into small sets of these variables called principal components. PCA was used to study the covariation of *H*(*e*_*i*_) calculated for** L**,** R**,** V**_**L**_, and** V**_**R**_ epochs. The first principal component is the one that accounts for as much of the variability in the data as possible, and each succeeding component, in turn, explains the subsequent amount of variance possible under the constraint that is orthogonal to the preceding components (i.e., uncorrelated). Factorial brain mappings were constructed to describe the results of the factorial analyses. These brain mappings were constructed by taking into loading values *f*_*j*_(*e*_*i*_) of each electrode *e*_*i*_ on each factor *F*_*j*_. There is no specific rule for selecting the variables that are significantly influential upon each *F*_*j*_ based on their loading values. However, in general, it is acceptable to focus attention upon those variables with loading values greater than 0.6. This is because factorial mappings here were built in color encoding electrodes as white if the loading was smaller than 0.6; otherwise, they were colored from green (loading 0.6) to dark blue (loading 1). Factorial mappings are proposed here to represent the activity of the neural circuits enrolled in a cognitive task because they condensed the information provided by the electrodes sampling this neural activity. This is because *H*(*e*_*i*_) measures the amount of information provided by *e*_*i*_ about spatial and temporal distributions of *s*_*l*_.

## 3. Results

Combined use of the different tools employed here to EEG analysis disclosed many different details of a widespread cortical activity during language perception supporting the hypothesis that speech processing is better understood in the context of DIPS theory. Due to the fact that the main purpose of the present paper is to investigate this relation and also for the sake of simplicity, description results in what follows will be focused on general findings supporting our main hypothesis, giving details for future reports.

### 3.1. Event Related Activity

No mistakes were made by the volunteers when they were asked to identify the correct figure associated with each text, independent of the type of task (reading or listening) or semantic category (fruits, instruments, or professions). The mean reaction time for this identification was 2010 ± 680 ms for listening tasks and 2360 ± 880 ms for reading tasks. These results are statistically similar (significance level *P* > 0.05).

**Figure 4:**
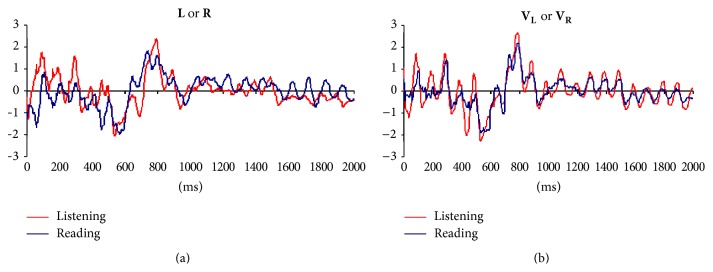
Grand averages for** L** and** R** (verbal phase) and** V**_**L**_ and** V**_**R**_ (visual phase) epochs for listening and reading tasks.

Figure 4 shows the grand averages calculated for** L**,** R**,** V**_**L**_, and** V**_**R**_ epochs. The temporal evolution of averaged EEG activity was very similar for both listening and reading tasks, as well as for verbal (**L** and** R**) and visual (**V**_**L**_ and** V**_**R**_) task phases. Pearson's correlation coefficients between these grand averages varied from 0.72 to 0.81.

Large negative components (N400) beginning approximately 300 ms (verbal phase) and 2300 ms (visual phase) after the task initiation and peaking approximately at 600 ms (verbal phase) and 2600 ms (visual phase) were clearly observed. At least two positive waves at (P100) 100 and P(300) 300 ms precede these negative components. Other large positive components (P600) beginning approximately at 600 ms (verbal phase) and 2600 ms (visual phase), peaking approximately at 800 ms (verbal phase) and 2800 ms (visual phase), and being followed by another negative component at approximately 900 ms (verbal phase) and 2900 ms (visual phase) were also clearly observed. Other negative (LN) components were identified at 1000/3000 ms, 1550/3600 ms, and approximately 1980/3980 ms. A sustained positive activity (LP) was observed from 1100/3100 to 1550/3500 ms, respectively.

### 3.2. Identified LORETA Sources

**Figure 5:**
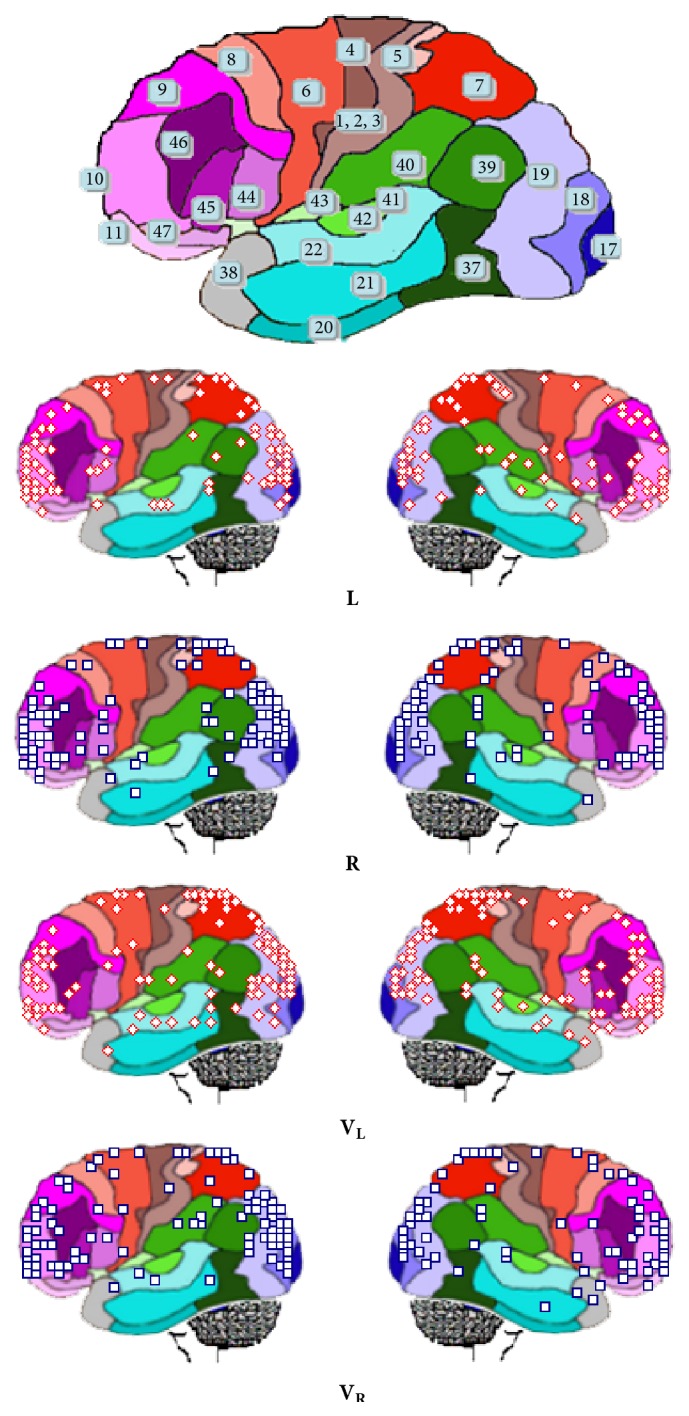
Spatial location of LORETA (**ILS**) identified sources of ERA calculated for** L**,** R**,** V**_**L**_, and** V**_**R**_ epochs. Numbers and color limits of Brodmann areas are shown just as approximate reference to** ILS** locations.

A total of 408 possible sources *s*_*l*_ of the averaged EEG (ERA) for epochs** L** and** V**_**L**_ were identified in 62 different cortical locations (*l*_*l*_) characterized by their Brodmann area number and anatomical structure because their calculated *Z* score was greater than 1.961. In addition, a total of 558 possible sources *s*_*l*_ of the averaged EEG for epochs** R** and** V**_**R**_ were identified in 57 different cortical locations.  Figure 5 shows the spatial distribution of the identified LORETA sources (**ILS**).

Of all identified *l*_*l*_, 50 sites were common to both listening and reading tasks; however, frequencies of the sources *s*_*l*_ located at these areas were different for** L**,** R**,** V**_**L**_, and** V**_**R**_ epochs. Locations *l*_*l*_ at BA 18 and BA 19 predominated and included the cuneus and middle occipital gyrus preferential sites. Areas BA 10 and BA 11 were second on the frequency concerning *l*_*l*_ and included the medial, middle, and superior frontal gyrus as preferential sites. Sources located at temporal structures in BA 20, BA 21, BA 22, and BA 37 were identified in all experimental epochs. Finally, BA 45, BA 46, and BA 47 hosted many *s*_*l*_. Some of the sources located at superior occipital gyrus at BA 19, fusiform gyrus at BA 37, and postcentral gyrus at BA 40 were specifically associated with reading.

**Figure 6:**
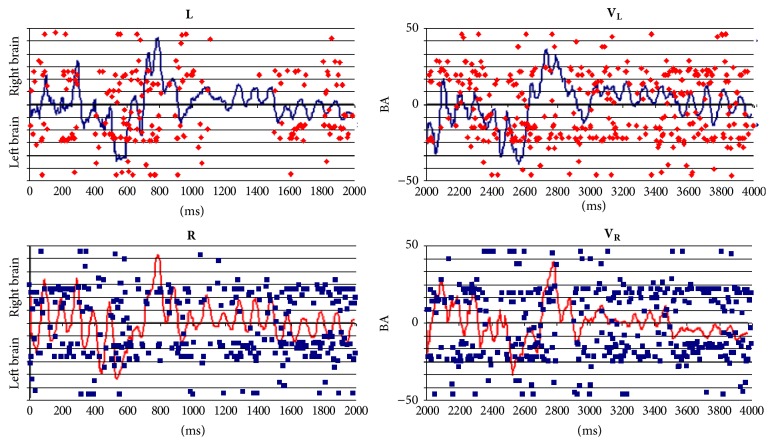
Temporal activation of LORETA sources identified during** L**,** R**,** V**_**L**_, and** V**_**R**_ epochs. BA: Brodmann area number. Time in milliseconds.

Figures 5 and 6 show** ILS**s spatial location and temporal distributions, respectively, during both the reading and listening tasks. It may be observed in Figure 5 that** ILS**s are widely distributed over the entire cortex, although they are preferentially located at BA 18 and BA 19 in the posterior brains and BA 10 and BA 11 in the frontal brain.  Figure 6 shows that these sources are continuously activated during the** L**,** R**,** V**_**L**_, and** V**_**R**_ epochs. Other cortical areas that are frequently and almost continuously activated are located at BA 44, BA 45, BA 46, and BA 46 in both hemispheres.

In addition, it may be observed that both spatial and temporal** ILS**s distributions are different when reading and listening tasks are compared and also when** L** or** V**_**L**_ epochs are compared to** V** or** V**_**R**_ epochs.

**Figure 7:**
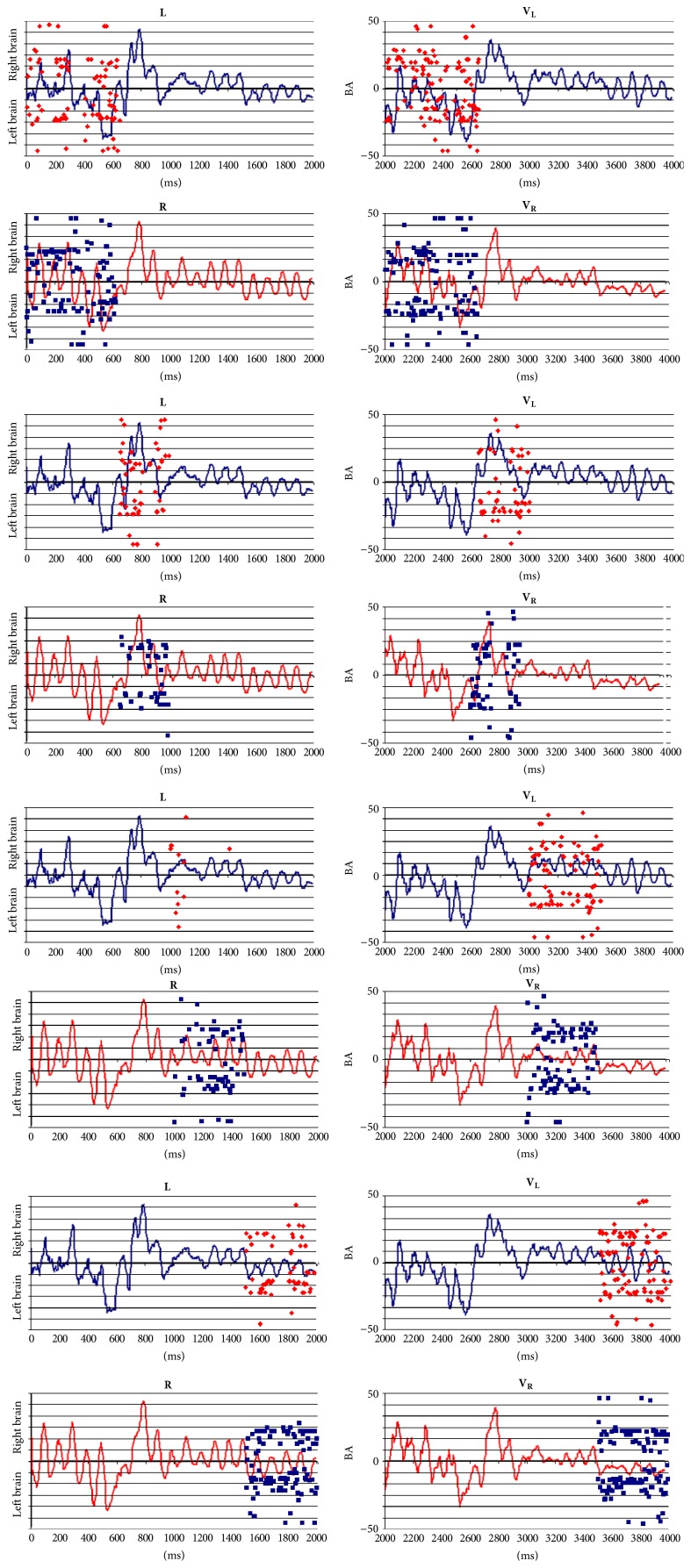
Temporal activation of LORETA sources identified epochs during 4 different time windows during** L**,** R**,** V**_**L**_, and** V**_**R**_ epochs.

Figure 7 shows** ILS **temporal distribution within time windows 0 to 600 ms, 600 to 1000 ms; 1000 to 1500 ms, and 1500 to 2000 ms. Components P100, P300, and N400 identified above occur in the first time window. Components P600, LN, and LP correspond to the other time windows, respectively. It may be observed that** ILS**s associated with these EEG components are different when both components are compared and reading and listening tasks are considered. Results in Figure 7 show that each of these components seems to be generated by specific cortical activities, or, in other words, different sets of** ILS**s provide the different EEG signatures identified in the grand averages calculated for the distinct EEG epochs.

### 3.3. Band Frequency Analysis

**Figure 8:**
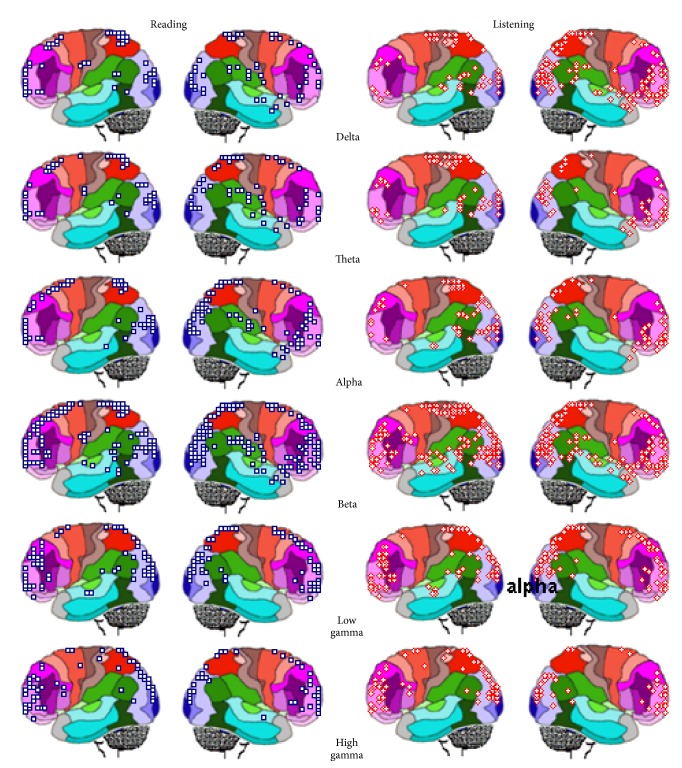
Spatial location of LORETA (**ILS**) identified sources during** L** and** R** for the classical band widths.

**Figure 9:**
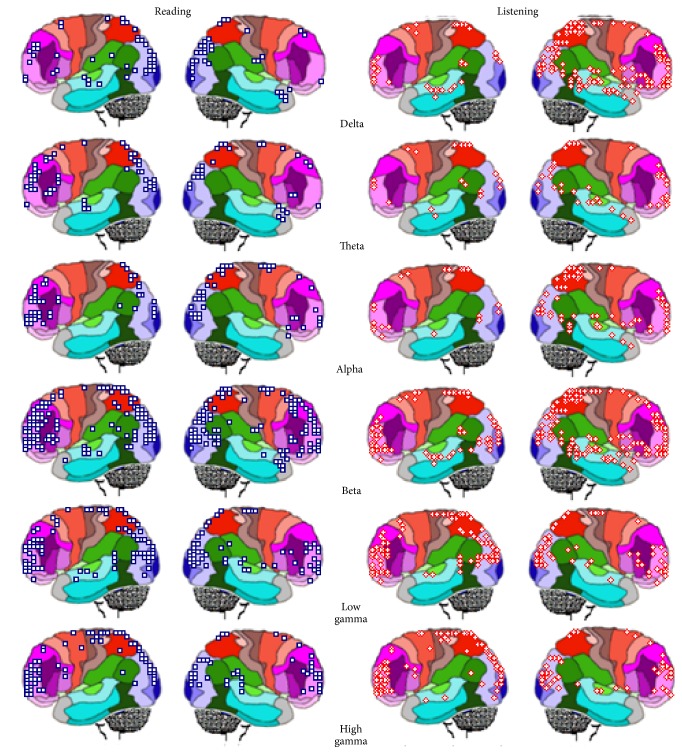
Spatial location of LORETA (**ILS**) identified sources during** V**_**L**_ and** V**_**R**_ for the classical band widths.

Figures 8 and 9 show the spatial location of** ILS** generating brain oscillations for the classical band frequencies: delta, 1 to 4 Hz; theta, 4 to 7 Hz; alpha, 8 to 13; beta, 14 to 35; low gamma, 30 to 60; and high gamma, 70 to 100.

**ILS**s for all band frequencies are distributed all over the cortex, predominating in the frontal pole (BAs 9, 10, 11, 46, and 47) and the occipital pole (BAs 17, 18, and 19), but their distribution is frequency and task sensitive. For example,** ILS**s located at BA 6 and BA 8 are specifically related to reading with activity being high at BA 8 for theta, beta, and alpha band frequencies and being high at BA 6 for beta and gamma bands. Besides, this pattern is much more evident during** L** and** R** than** V**_**L**_ and** V**_**R**_ epochs.

Activity at BA 40 during verbal epochs** L** and** R** predominates for theta to beta band frequencies in listening in contrast to reading, for left in comparison to right hemisphere, and for verbal in contrast to visual epochs. In addition, activity at BA 22 predominated for listening compared to reading mostly in case of theta and alpha band frequencies and at right compared to left hemisphere (Figure 8).

Activity at BA 40 predominates at both hemispheres during visual epochs** V**_**L**_ and** V**_**R**_ for almost all band frequencies in case of listening compared to reading (Figure 9).

Finally, activity at BA 10 predominates at both hemispheres for beta and gamma band frequencies for all experimental epochs** L**,** R**,** V**_**L**_, and** V**_**R**_.

### 3.4. Principal Components Analysis

Table 1 shows the results for PCA analysis when the amount of information *H*(*e*_*i*_) provided by each electrode *e*_*i*_ about the different** LIS**s was calculated for all experimental epochs** L**,** R**,** V**_**L**_, and** V**_**R**_ and both reading and listening (**A**) and for each one of these activities (**R** and** L**, resp.).

**Figure 10:**
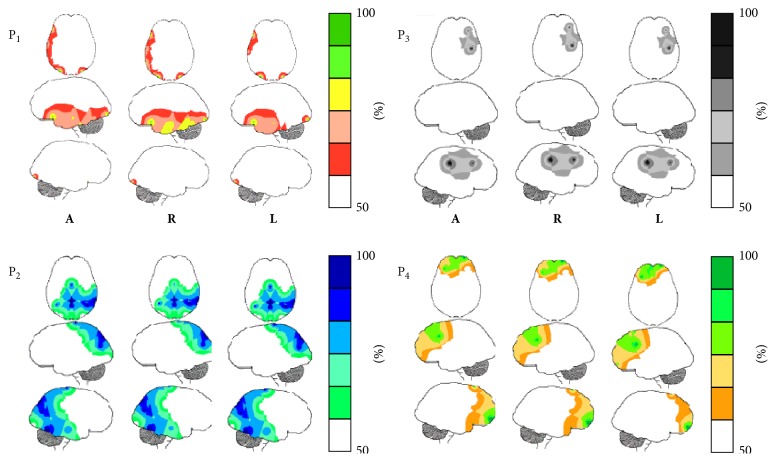
PCA mappings (P_1_ to P_4_) for the loading values on each factor as shown in Table 1.** A**: all tasks;** R**: reading tasks;** L**: listening tasks. Loading values smaller than 0.5 were color-encoded in white and those greater than 0.5 were color-encoded according to the displayed scales.

This analysis disclosed the existence of 4 different factors (P_1_ to P_4_) with eigenvalues greater than 1 that accounted for around 80% of *H*(*e*_*i*_) covariance. These results show that factors P_1_ to P_4_ are robust from the statistical point of view. The loading values on each of these factors were used to build the PCA mappings shown in Figure 10, where those electrodes having loading values greater than 0.6 are shown in green to dark blue and those having loading values smaller than 0.6 are shown in white. It can be observed that these mappings are very similar for both reading (**R**) and listening (**L**) tasks, as well as when these activities were considered together (**A**).

Factor P_1_ is composed of electrodes F7, T3, T5, O1, and O2 for both** A** and** R**, but T5 is missing in case of** L**; factor P_2_ is composed of electrodes CZ, OZ, P3, P4, PZ, T4, and T6; factor P_3_ is composed of electrodes C4 and F4; and factor P_4_ is composed of electrodes F3, FP1, FP2, and FZ. It is interesting to remark that P_1_ is predominately composed of left hemisphere electrodes while P_4_ is composed of right hemisphere electrodes.

### 3.5. LORETA and PCA Mappings

**Figure 11:**
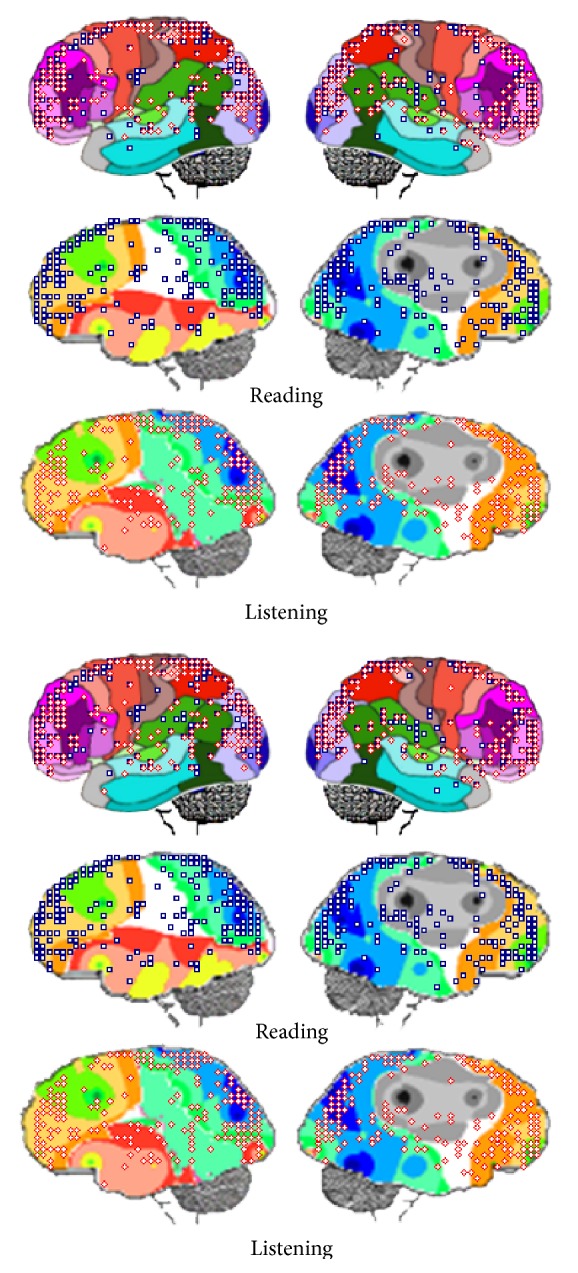
LORETA sources associated with PCA mappings P_1_, P_2_, and P_4_ for both listening and reading and epochs** L** and** R** (above) and** V**_**L**_ and** V**_**R**_ (above).

PCA patterns are proposed to disclose set of electrodes that provide information about neurons that enroll together to carried defined computations [44]. This is because EEG activity recorded by the different electrodes is mostly determined by nearby sources. Figure 10 shows spatial** ILS**s distribution superimposed over PCA mappings for identification of the possible sources locations *l*_*l*_ associated with each PCA mapping shown in Figure 11.

Inspection of Figure 11 reveals that** ILS**s located at left BAs 22, 42, 43, 44, and 45 are located near the electrodes loading on pattern P_1_ that is composed mostly of left hemisphere electrodes. In addition, activity of** ILS**s identified at lower locations of BAs 37, 39, and 40 may have also been recorded by P_1_ electrodes. Finally, it seems that** ILS**s located at lingual and inferior occipital gyri may have also contributed to EEG activity recorded by P_1_ electrodes.

Activity recorded by P_2_ electrodes may be influenced by** ILS**s bilaterally located at BAs 7, 18, and 19, as well as at upper locations of 37, 39, and 40. In addition, many cortical neurons associated with P_1_ at the left hemisphere are close to right electrodes composing P_2._

Pattern P_3_ is the simplest one and is composed only of right hemisphere electrodes that may have recorded activity of neurons located at right BAs 4, 5, and 6. Finally, electrodes loading in pattern P_3_ may have recorded activity of** ILS**s located bilaterally at BAs 4, 6, 8, 9, 10, and 46.

## 4. Discussion

Various techniques for EEG analysis were used to study the dynamics of neural activity associated with listening to spoken language and reading written texts. The results clearly show the high complexity of the cerebral activities associated with these tasks that involved very complex temporal activation of a large number of sets of neurons (EEG sources *s*_*l*_) widely distributed over the entire cortex. In this context, language processing is best understood as the result of distributed processing with different sets of neurons *s*_*l*_ taking charge of specific analyses and exchanging information about them. Thus, speech understanding and production are the result of a cooperative action among this large number of *s*_*l*_, rather than dependent on the capability of neurons located at a unique and specific brain area (e.g., [17, 22, 23, 32]).

The high temporal EEG sensitivity is the keystone to characterize the complexity of the temporal information exchange between the large numbers of neurons composing the distinct** ILS**s disclosed by LORETA and PCA analyses. However, the cerebral activity recorded with EEG is restricted to that taking place at the cortical level, and this is a restriction to present results and hypotheses they may support. The discussion that follows must be understood under these hypotheses and constraints.

### 4.1. Event Related Activity and Linguistic Decoding

One interesting finding of the present research concerns the similarity (Figure 4) of EEG grand averages or Event Related Activity (ERA) obtained for both** L** and** R** epochs and, most strikingly, the persistence of this EEG pattern for the electrical activity recorded during** V**_**L**_ and** V**_**R**_ epochs.

ERA associated with verbal decoding of linguistic information is predominately characterized by negative activity peaking approximately at 600 ms and followed by a positive activity peaking approximately at 800 ms. This pattern is in clear agreement with the literature when discussing the role played by N400 and P600 components in the syntactic and semantic processing associated with sentence comprehension during** L** and** R** epochs [19, 32, 36–38, 42]. Here, the N400/P600 signature of speech processing started at approximately 300 ms and ended at approximately 800 ms. This classical N400/P600 complex was followed by a negative component peaking around 900 ms, a sustained positive activity from 1000 to 1500 ms, and another clear negative component around 1500 ms.

Texts used in present experiments have two different syntactical components: triggering and solving sentences. Task solution requires selection of one among five possible figures providing the semantics to name (**N?**) a fruit, tools, or profession. Thus, task solution requires an amount of 2.32 bits of information. The triggering component provides most of the required information, in general reducing solution to choice among two alternatives or to 1 bit. We propose, here, that the N400/P600 complex is associated with triggering component processing and the N900/P1000/N1500 complex processes the solving component besides integrating both triggering and solving information to identify** N?**.

The intriguing fact here is the persistence of the same ERA pattern during** V**_**L**_ and** V**_**R**_ epochs, when predominance of visual processing of figures carrying out text meaning was expected. However, this may be easily understood if it is assumed that verbal information obtained during** L** and** R** epochs was held in working memory during visual inspection and analysis of the figures during** V**_**L**_ and** V**_**R**_ epochs [10]. If this hypothesis is accepted, then verbal information encoded in the N400/P600 and N900/P1000/N1500 complexes guided the concurrent visual processing for visual recognition of the previously recognized** N? **or for finally identifying the semantics of** N?**. In this context, the task solution was provided by matching each piece of the given verbal information and their visual counterpart (e.g., name color and color, action name and action) and/or associating the verbal name with a recognized image (e.g., hammer, waiter, and orange). In this line of reasoning, both common and distinct cortical sources  *s*_*l*_ should contribute to the recorded ERA components for all studied EEG epochs as observed in the present study (e.g., Figure 7).

### 4.2. LORETA Discloses the Neural Complexity of Linguistic Processing

LORETA identified a large number (greater than 400) of sources *s*_*l*_ located at more than 60 different cortical areas, as defined by their Brodmann area number and anatomical structure (Figure 5). The majority of these cortical areas were the same for the different activities involved in** V, R, V**_**L**_, and** V**_**R**_ epochs. However, both the temporal and spatial locations of these *s*_*l*_ were different for each of these epochs. This means that although EEG activity recorded during different experimental epochs was generated by sources in approximately similar locations (BAs), the dynamics (frequency and intensity of activation) were different. This agrees with the proposition by Price [17] who, after reviewing language fMRI literature for the last 20 years, concluded that “the different language functions are not localized in specific brain regions, but they were distributed across networks of regions with each area making a specific contribution to performance of the task which depends on its connections to other areas in a parallel distributed hierarchy.”

Neuronal oscillations define short temporal windows for flexible communication between widely distributed neuronal ensembles [17, 33]. Although broad synchronization in distributed processing systems is dependent on the action of specific circuits, as in thalamus-cortical synchronization, short term communication is dependent on interconnections between the sets of neurons located in different brain areas supporting transient functional couplings [17].

Here, we used a 100 ms time window to study the temporal evolution of neuronal oscillation associated with listening and reading by using LORETA to identify the sources *s*_*l*_ of such oscillations.** ILS**s were different for each language activity and were also distinct when right and left hemispheres were considered. The frequencies at which *s*_*l*_ were located at left, but not at right, BAs 6 and 7 and BAs 21, 22, 37, 39, and 40 predominated for reading compared to listening (Figures 8 and 9). These results support the proposal that short term communication among neurons involved in language processing is supported by transient functional coupling between specific sets *s*_*l*_ of neurons located at the different cortical areas [17]. This implies that information exchange between neurons followed different patterns for listening and reading, even if these activities enrolled cells located at the same cortical areas. Such findings stress the distributed character of speech understanding.

### 4.3. *H*(*e*_*i*_) PCA Discloses Four Patterns of Neural Activity

Complex *s*_*l*_ temporal and spatial activation instances were observed for all experimental epochs** L**,** R**,** V**_**L**_, and** V**_**R**_ (Figures 6 and 7). Because of this, the amount of information *H*(*e*_*i*_) from each electrode *e*_*i*_ about the different** ILS**s was calculated, and PCA was used to study the possible patterns of *H*(*e*_*i*_) covariation. This analysis revealed four different patterns (P_1_ to P_4_) of EEG activity associated with language processing that explained 80% of data covariance, as observed in other studies [22, 24, 44, 50]. Eigenvalues and loading values associated with these patterns were high, indicating robust results. The PCA mappings in Figure 10 show the electrodes with loading values greater than 0.6 in the different PCA factors and clearly suggest that four different neural circuits were involved in decoding the descriptive texts used in the present experiments.

Pattern P_1_ is composed of electrodes F7, O1, O2, and T3 in all conditions illustrated in Table 1, and Figure 10 includes T5 in** R**. It is proposed here that the pattern P_1_ describes the covariation of the amount of information *H*(*e*_*i*_) provided by the above electrodes about the sources** ILS**_4_ that are located in the left temporal BAs 20 to 22 (which includes Wernicke's area BA 22), the left frontal BAs 44 to 47 (which includes Broca's area), and other left structures, such as the angular gyrus (BA 39), the fusiform gyrus BA 37 (also known as visual word form area), and the supramarginal gyrus (BA 40), which are all involved with language processing. The comparison of mapping P_1_ for listening and reading activities differs because electrode T5 is not included in the listening case. As a consequence, it may be said that these sources *s*_*l*_ located at BAs 37, 38, and 39 are less influential on processing of oral language in comparison to written language. Another interesting finding was that activity at BAs 6 and 8 clearly differentiates reading from listening task. Activity of these areas may be related to eye control required for reading [17]. PCA pattern P_1_ shows that neural activity at these locations is coherently organized with the purpose of taking into account the analysis of the linguistic content of the oral or written texts.

This hypothesis is supported by many studies reported in the literature. For example, Brennan and Pylkkänen [2] showed that the anterior temporal activity for sentences decoding begins to increase approximately 250 ms after sentence onset. They also observed increased activation in a network of other brain areas, extending across the posterior temporal, inferior frontal, and ventral medial areas. In addition, Kunii et al. [51] confirmed that high gamma activity at the inferior frontal and middle temporal gyrus was positively correlated with language decoding. The most striking finding was the different temporal dynamics of these different brain regions, with frontal lobe showing longer-lasting activity, while activation in the temporal lobe quickly declined. Goto et al. [7] also showed that transient power increases in the theta band occurred first in the bilateral occipital cortices and then rapidly propagated to the left temporal-occipital areas, the left inferior and middle frontal gyrus, the bilateral medial prefrontal cortices, and finally the left anterior temporal cortices, which possibly reflects a serial cognitive process.

Confirming that linguistic decoding depends on the activity at different cortical areas, Shirahama et al. [52] studied electrical activity associated with silent reading and identified a dipolar source approximately 100–250 ms in the fusiform gyrus and another one approximately 300–500 ms in the vicinity of the superior temporal gyrus or the angular gyrus in the right and left hemispheres, which may be associated with the reading phonological pathway [53]. Adding to this, Levy et al. [54] proposed that reading linguistic elementary analysis is confined to the activation of the bilateral posterior regions, but linguistically complex stimuli additionally recruit the left hemispheric anterior regions, raising the hypotheses of gradual bilateral-to-left and a posterior-to-anterior recruitment of reading related areas. In the same line of reasoning, Pugh et al. [55] assumed the existence of two reading circuits in the left hemisphere (LH) posterior systems: a dorsal (temporal-parietal) circuit and a ventral (occipital-temporal) circuit. The dorsal circuit predominates at first and, in conjunction with premotor systems, is associated with analytic processing necessary for learning to integrate orthographic with phonological and lexical-semantic features of printed words. The ventral circuit constitutes a fast, late-developing, word form system, which underlies fluency in word recognition. As we discussed above, sources located at BA 37 (both at MTG and ITG) may be associated with the word visual form area, a key element in the reading lexical pathway [53].

Pattern P_2_ is composed of electrodes CZ, OZ, P3, P4, PZ, T4, and T6 in all conditions (see Table 1 and Figure 10), and it is proposed, here, that it describes the covariation of the amount of information *H*(*e*_*i*_) provided by these electrodes about the sources located on the right at BAs 20 to 22, BAs 37 to 40, and BAs 44 to 47, as well as at BAs 1 to 8 and BAs 18 to 19 in both hemispheres. Due to this, it is proposed that pattern P_2_ is associated with neural circuits in charge of the semantic decoding of the verbal and visual information. This proposal agrees with recent literature about text understanding. Following this line of reasoning, Xu et al. [56] studied decoding of linguistically matched sets of texts when these were differentially presented as random word lists, unconnected sentences, and coherent narratives. They found that the same stimuli presented as narratives evoked robust responses in extrasylvian areas within both hemispheres, including precuneus, medial prefrontal, and dorsal temporal-parietal-occipital cortices. The right hemisphere was increasingly active as contextual complexity increased and was maximal at the narrative level. Furthermore, brain activity was dynamically modulated as subjects processed different narrative segments: left hemisphere activity was more prominent at the onset, and right hemisphere was more prominent at the resolution of a story, at which point it may support coherent representation of the narrative as a whole.

P_3_ is composed of electrodes C4 and F4 T6 in all conditions (see Table 1 and Figure 10), and it is proposed here that it describes the covariation of the amount of information *H*(*e*_*i*_) from these electrodes about the sources located in BAs 1 to 8, which are constituents of the somatosensorial and motor cortices. In this way, pattern P_3_ would be proposed to disclose neural integration related to mouse control for reaching visual targets representing text semantic decoding.

Finally, P_4_ is composed of electrodes F3, FP1, FP2, and FZ T6 in all conditions (see Table 1 and Figure 10). It is proposed here that it describes the covariation of the amount of information *H*(*e*_*i*_) from these electrodes about the sources located in BAs 9 to 11 and BAs 44 to 47, which are classically assumed to be in charge of executive functions.

This hypothesis is supported by studies such as Hald et al. [9] that explored the nature of the oscillatory dynamics in the EEG of subjects reading sentences that contain semantic violation. The authors found that a wavelet-based time-frequency analysis revealed a theta band power increase during an interval of 300–800 ms after critical word onset, at temporal electrodes bilaterally for both sentence conditions, and over the midfrontal areas for the semantic violations only. In addition, Cooper et al. [57] manipulated participants' interpretations of texts by asking them to focus on action-space or time-related features while listening to identical short stories. They observed that activity in the posterior left IFG (pars opercularis) showed different activity levels for the three conditions. However, a population coding analysis demonstrated similar distributions of activity across conditions. They concluded that while the gain of the response in the pars opercularis was modulated, its core organization was relatively invariant across the experimental conditions. Their findings suggested that a substantial source of variance in neural activity during language comprehension emerges from the internally driven, information-seeking preferences of listeners, rather than the syntactic or semantic properties of a text. In the same line, Metusalem et al. [14] acknowledged that real-world events play an important role in guiding online language comprehension and proposed that generalized event knowledge activation contributes to mental representations of described events; it is also immediately available to influence language processing and likely drives linguistic expectancy generation.

### 4.4. Listening and Reading

Available information from the literature shows that both listening and reading recruit neurons in many different cortical areas (e.g., see the reviews [17, 53]), such that competence on reading cannot be assigned to single cortical areas, such as the Visual World Form Area reported by many authors. At least two different large neural circuits have been implicated in reading and named the Phonological and Lexical Pathways (e.g., [53]). Levy et al. [54] reported the existence of bilateral-to-left and posterior-to-anterior recruitment of reading related areas that result from the increase in the stimuli's linguistic processing load and that reflects reading process activities, such as visual analysis, orthographic encoding, and phonological decoding. They claimed that their findings clearly establish the notion of gradual spatial-functional recruitment of reading areas evidencing a robust and staged link between the level of linguistic processing, the spatial distribution of brain activity, and its information trafficking. Despite these differences between listening and reading, the literature also points to the fact that these two linguistic activities enroll neurons in many common areas (e.g., [17]) because syntactic and semantic processing remain the same, independent of the sensory source of verbal information. In addition, we may observe that reading usually recalls the oral encoding of words and vice versa. An interesting finding of the present study is the enrollment of BAs 6 and 8 in reading but not in listening that as far as we know was not specifically reported in the literature. Present results as mentioned below are in agreement with this distributed processing of reading and listening.

The temporal evolution of the averaged EEG activity was very similar for both listening and reading tasks and verbal tasks (Figure 4). Approximately 400 possible EEG sources *s*_*l*_ were identified for listening, and approximately 550 sources were identified for reading. Most sources were located in the same cortical areas identified by their Brodmann's area number and anatomical structure (Figure 5). However, the frequency they were identified with at these locations differed when reading and listening were compared (Figures 6 and 7). The most important differences were observed for BAs 1 to 7 and BAs 18 and 19.

TVCS analysis also showed that although many of the sources *s*_*l*_ identified for each studied frequency are located at the same cortical areas, the locations of** ILS**s for each language activity in the right and the left hemispheres were different (Figures 8 and 9). For example, the frequencies at which *s*_*l*_ were located at the left, but not at the right, BAs 6 and 8 were greater for reading compared to listening. In the same way, *s*_*l*_ were more frequent at the left, but not at the right, BAs 21, 22, and 37 for reading compared to listening. The source location at the superior frontal gyrus at BA 10 predominated in the left hemisphere compared to the right one. Finally, the temporal and spatial *s*_*l*_ distributions were different for listening and reading. Although the majority of *s*_*l*_ were identified at the same location, they were, in general, activated at different times when listening and reading activities were considered.

In contrast with the above distinctive characteristics of listening and reading neural processing, *H*(*e*_*i*_) PCA brain mappings were very similar for both reading (**R**) and listening (**L**) tasks. However, the pattern P_1_ did not include electrode T5 in listening activities. LORETA analysis associated BAs 37, 38, and 39 with this electrode (Figure 11). As a consequence, it may be said that the influence of sources *s*_*l*_ located at BAs 37, 38, and 39 is less influential on the processing of oral language in comparison to written language.

*H*(*e*_*i*_) PCA shows that despite the temporal and spatial complexity of the neural dynamics involved in listening and reading, the activity of the recruited neurons may be understood by taking into account a small number of high cognitive functions involved in theanalysis of the linguistic content of the oral or written texts as disclosed by pattern P_1_, the semantic decoding of the verbal and visual information as disclosed by pattern P_2_, and the executive functions as disclosed by pattern P_1_ and involved in organizing and integrating the above syntactic and semantic analyses.

## 5. Conclusions

The set of tools for EEG analysis used in the present study allowed us to obtain important information about the temporal and spatial relationships between large sets *s*_*l*_ of neurons involved in language processing. The electrical activities *v*_*i*_(*t*) generated by these *s*_*l*_ were recorded by the electrodes placed according to the 10/20 protocol. LORETA analysis showed that different sets (**ILS**) of *s*_*l*_ were associated with ERA and BFA sources during text reading, listening, and decoding. The information *H*(*e*_*i*_) provided by the electrodes about *s*_*l*_ was calculated taking into consideration the linear correlation between *v*_*i*_(*t*) recorded by the different electrodes. The temporal and spatial activation of these *s*_*l*_ followed very complex dynamics which was summarized by *H*(*e*_*i*_) PCA analysis into four different patterns of EEG activity. Pattern P_1_ is associated with circuits in charge of text linguistic processing; pattern P_2_ is associated with neural circuits in charge of determining the semantic value of the texts; pattern P_3_ is associated with neural circuits integrating verbal and visual information required by selecting the adequate picture to each text; and pattern P_4_ is associated with neural circuits in charge of the task's executive control. In this context, language processing is best understood as the result of distributed processing with different sets of neurons taking charge of specific analyses and exchanging information about these analyses. Thus, the solution of the cognitive task (listening, reading, writing, speaking, etc.) in question is the result of a cooperative action of this large number of different neural sets, rather than depending on the capability of neurons located at a unique and specific brain area.

Although LORETA provides an acceptable solution for the inverse problem of recovering sources that generated the recorded EEG, it must be remembered that this solution is restricted to activity recorded from cortical but not subcortical structures. Therefore, it must be kept in mind that other techniques for recording activity must be used in combination with EEG if investigation aims to study subcortical neurons, too.

## Figures and Tables

**Table 1:** Factor loading values disclosed by *H*(*e*_*i*_) PCA taking into consideration all EEG epochs (**A**) and all EEG epochs for reading (**R**) and listening (**L**) tasks.

	**A**				**R**				**L**			
	P_1_	P_2_	P_3_	P_4_	P_1_	P_2_	P_3_	P_4_	P_1_	P_2_	P_3_	P_4_
C3	0.42	0.58	0.13	0.32	0.40	0.54	0.14	0.37	0.42	0.60	0.12	0.29
C4	0.28	0.21	*0.85*	0.15	0.29	0.25	*0.83*	0.16	0.27	0.20	*0.86*	0.15
CZ	0.04	*0.80*	0.08	0.43	0.01	*0.75*	0.08	0.52	0.06	*0.82*	0.07	0.40
F3	0.35	0.14	0.08	*0.81*	0.41	0.12	0.08	*0.80*	0.33	0.16	0.09	*0.81*
F4	0.10	0.36	*0.78*	0.37	0.11	0.35	*0.79*	0.39	0.09	0.36	*0.78*	0.37
F7	*0.76*	0.19	−0.01	0.44	*0.76*	0.19	0.06	0.43	*0.76*	0.20	−0.03	0.43
F8	0.25	0.44	0.27	0.58	0.21	0.39	0.32	*0.62*	0.27	0.46	0.25	0.56
FP1	0.41	0.01	0.46	*0.68*	0.47	0.00	0.47	*0.63*	0.40	0.02	0.46	*0.69*
FP2	0.17	0.07	0.18	*0.83*	0.21	0.04	0.20	*0.84*	0.17	0.09	0.17	*0.82*
FZ	−0.02	0.49	0.20	*0.71*	0.04	0.44	0.20	*0.74*	−0.04	0.51	0.20	*0.69*
O1	*0.81*	0.22	0.40	0.13	*0.80*	0.25	0.41	0.12	*0.82*	0.21	0.40	0.13
O2	*0.76*	0.35	0.34	0.11	*0.74*	0.39	0.32	0.10	*0.76*	0.33	0.36	0.11
OZ	0.32	*0.64*	0.38	0.11	0.36	*0.63*	0.37	0.12	0.29	*0.64*	0.39	0.11
P3	0.34	*0.79*	0.33	0.04	0.35	*0.80*	0.30	0.08	0.32	*0.79*	0.33	0.02
P4	0.28	*0.80*	0.41	−0.01	0.28	*0.82*	0.39	−0.02	0.27	*0.80*	0.42	0.00
PZ	0.17	*0.81*	0.30	0.09	0.19	*0.83*	0.26	0.08	0.16	*0.81*	0.31	0.11
T3	*0.68*	0.49	0.08	0.27	*0.67*	0.50	0.05	0.29	*0.68*	0.49	0.09	0.26
T4	0.20	*0.74*	0.07	0.34	0.16	*0.73*	0.07	0.41	0.22	*0.74*	0.07	0.31
T5	*0.62*	0.43	0.11	0.30	*0.71*	0.32	0.10	0.30	0.58	0.48	0.11	0.30
T6	0.40	*0.83*	0.01	0.15	0.37	*0.84*	0.04	0.17	0.41	*0.82*	0.00	0.15
Expl.Var	3.82	5.79	2.53	3.64	3.97	3.86	2.49	3.86	3.75	5.93	2.56	3.50
Prp.Totl	0.19	0.29	0.13	0.18	0.20	0.19	0.12	0.19	0.19	0.30	0.13	0.17
